# Editorial: Neuronal Mechanics and Transport

**DOI:** 10.3389/fncel.2016.00001

**Published:** 2016-01-26

**Authors:** Kyle E. Miller, Daniel M. Suter

**Affiliations:** ^1^Department of Integrative Biology, Michigan State UniversityEast Lansing, MI, USA; ^2^Department of Biological Sciences, Bindley Bioscience Center, Purdue UniversityWest Lafayette, IN, USA

**Keywords:** neuronal mechanics, force, neuronal development, stiffness, glia, neuronal transport, axonal elongation, neuronal morphology

Past research on nervous system development was largely centered on the role of molecules and biochemical signaling cascades. However, recent biophysical work has clearly demonstrated the additional importance of forces in shaping neuronal morphology and driving intracellular transport as well as the role of stiffness for cellular behavior. The research topic “Neuronal Mechanics and Transport” brings together related research articles and reviews with the long-term goal of fostering a community of scientists interested in this interdisciplinary topic.

## The shaping of neurons, glia, and the brain

The importance of forces in shaping the morphology of the nervous system spans scales ranging from the whole brain down to individual cells and involves both neurons and glia. Budday et al. focus on cortical folding. They briefly review the developmental biology of the human brain and then discuss modeling and experimental work designed to elucidate the mechanisms underlying cortical folding. They emphasize that local differences in stiffness, growth rate, and cellular force balance during normal development lead to the characteristic pattern of folding and discuss how diseases that impact these underlying processes lead to either increases or decreases in the number of folds. Bollmann et al. investigated durotaxis in microglia using traction force microscopy (Bollmann et al.). Using experimental and theoretical analysis, they make a compelling argument that microglia preferentially migrate toward stiffer regions because they take larger steps in the direction of the stiffer region. In turn Schwann cells are important for myelinating neurons in the peripheral nervous system. Like neurons, they have an extended geometry and locally synthesize proteins in response to local signals and protein demand. Love and Shah monitored ribosomal transport in Schwann cells by expressing a GFP tagged version of the ribosomal L4 subunit and then developed a rate kinetics model of ribosomal transport. They found that initially levels of transport were high, but then declined as Schwann cells began myelination. Together these studies are important because they remind us that glia are as important as neurons when considering the mechanics of nervous system.

## Controlling axonal and dendritic length, branching, and diameter

Controlling local protein production and long distance transport of material is a major engineering challenge for neurons. A recent idea proposes that neurons can sense the length of axons and in turn control the rate of protein production. This leads to the obvious question of how. Bressloff and Karamched build on the idea, first developed in yeast, that frequency information (i.e., regular variations in the concentration of signaling proteins) is used to regulate the activity of gene networks. They propose that as axons lengthen, the frequency of an encoded signal drops, which in turn alters the rate of material production. This work is important because it points out that as axons lengthen the precision of axonal length measurement may decrease, and it highlights the need for future experimental work. Directly related to the problem of length control is the question of how process branching is regulated. Sholl analysis is an important tool that assesses branching by measuring the number of neuronal processes that pass through concentric circles spaced at fixed internals from the cell body. O'Neill et al. compared Inside-Out, Root-Intermediate-Terminal, and Tips-In Sholl analysis while characterizing the effect of cytosolic PSD-95 interactor (cypin) on the patterning of the dendritic arbors of hippocampal neurons. They found that each approach can detect large changes in branching, but they have regional differences in their sensitivity in detecting subtle variations. They suggest that standard Sholl analysis might be improved by integrating these approaches and by making their use more transparent in software used by biologists. Finally, axons have the remarkable geometrical property of having a relatively uniform diameter over distances that can be as long a meter in humans. How diameter is consistently maintained is especially puzzling as necking (i.e., local thinning) of materials typically occurs in response to large deformations. Two of the founders of the field of neuronal mechanics Heidemann and Bray, propose the novel theory that tension may locally induce breakage and the local compaction of neurofilaments and microtubules. As a result, the transport of cytoskeletal elements along the axon is locally inhibited and material accumulates and allows the axon to return to its original diameter in regions that are locally thin. Together these studies point out the importance of understanding the relationship between neuronal geometry, mechanics, and transport.

## Brain strain and electrical excitability

Most studies on neurons are typically concerned with either mechanical or electrical behavior, yet it has become clear that electrical and mechanical properties in neurons are related to each other. Fan et al. combined an innovative use of flavoprotein auto-fluorescence to track neuronal activity and controlled stretching of mouse brain slices. They demonstrate stretch-induced deformations of neuronal tissue significantly increases electrical excitability of neurons. Drapaca and Drapaca proposes an innovative electromechanical model of neurons that takes into account their viscoelastic and electrical properties as described by a Kelvin-Voigt model and the Hodgkin-Huxley equations. In turn, to test if slow stretch-induced axonal growth leads to changes in electrical activity as seen in rapid deformation, Loverde and Pfister grew rat sensory neurons in axon stretch-growth bioreactors and assessed neuronal activity using whole-cell patch clamp recording, free calcium imaging, and morphological analysis. They found no evidence of large changes in response to slow stretching, which suggests normal axonal lengthening driven by forces is distinct from traumatic axon injury. Together these studies highlight the importance of considering both the mechanical and electrochemical activity in neurons and the need for future studies on this complex problem.

## Signaling and molecular mechanisms of axonal elongation

In addition to mechanical forces, biochemical signaling pathways are critical in guiding growth cones correctly to their final destinations. Semaphorin 3A (Sema3A) is an important repulsive guidance cue that modulates the activities of Rac, Cdc42, and RhoA. The spatial and temporal pattern of activation and inactivation of these Rho family GTPases is poorly understood in growth cones. Using FRET microscopy in combination with an innovative method for focally applying Sema3A, Iseppon et al. analyzed how the activities of Cdc42 and RhoA change in response to Sema3A. As expected, they found that Sema3A leads to growth cone collapse, progressive activation of RhoA and overall deactivation of Cdc42. Interestingly, they found waves of Cdc42 activation propagate away from local sites of Sema3A application. An additional level of signaling control in neurons occurs through reactive oxygen species (ROS). While it is undoubted that aberrantly high levels of ROS are damaging, very low levels also disrupt normal neuronal functioning. Wilson and González-Billault review redox balance in neurons with a focus on the complex relationship between high, low, and normal levels of ROS, cellular signaling, cytoskeletal dynamics, vesicle trafficking, axonal elongation, and cognitive function. Of course these signaling pathways must target cytoskeletal elements. Microtubule plus end tracking proteins (+TIPs) bind to the plus ends of microtubules where they regulate assembly, the interaction of microtubules with the actin cytoskeleton and cell signaling. Bearce et al. review recent discoveries of well-established +TIP proteins such as CLASP and EB proteins as well as more newly recognized +TIP proteins XMAP215, TACC3, spectraplakins, and navigators. The review highlights the importance of +TIP proteins in growth cone guidance and the need for more sophisticated tools to study +TIP function including photo-manipulatable proteins and super resolution microscopy. Together, these studies point out the importance for future work that links cellular signaling and neuronal mechanics.

## Neuronal mechanics and forces are dynamic

An important part of understanding neuronal mechanics is describing force generation in time and space. Over the past years, there has been a surge in the development of technologies that now allow precise control over the physical environment that growth cones navigate, the application of forces to neurons and measurement of the forces they produce. Kerstein et al. provide an excellent overview of the mechanochemical regulation of growth cone motility that covers the generation of traction forces, the molecules important for force sensing, durotaxis, and the interaction between physical and chemical guidance cues. Kilinc et al. expand on this by reviewing newly developed technologies for controlling substrate stiffness, topology, and the chemical extracellular environment in which growth cones navigate. Polackwich et al. specifically investigated the traction forces generated by rat sensory neurons grown in polyacrylamide hydrogels. They showed that growth cones generate force dipoles on the substrate that are similar to those produced by migrating cells, but differ in that the force vectors create a net force that pulls the substrate rearwards and the axon forward. In addition, they noted large fluctuations in force generation that vary with a timescale of tens of seconds. In light of previous studies, they suggest these fluctuations arise as the result of the dynamic formation and disassembly of adhesions to the substrate.

Molecular motors such as kinesin, dynein, and non-muscle myosin II are critical for neuronal force generation. An important new tool for investigating dynein is the novel selective cell permeable dynein inhibitor ciliobrevin. Roossien et al. review its use of in the context of neuronal growth. They provide a brief overview of dynein function, discuss the puzzling question of why disruption of dynein inhibits kinesin (and vice versa) and the need for studies that elucidate the mechanism of action of ciliobrevin. They conclude with a summary figure that suggests dynein contributes to axonal elongation through the well-accepted long-range transport of cytoskeletal elements, organelles, and signaling molecules and in addition through the generation of forces that push the microtubule array forward. Building on the idea that dynein generates extensile forces on the microtubule array along the axon, Jakobs et al. modeled microtubule bundling and the forces associated with microtubule motors in the context of axonal elongation. Their work suggests that unipolar motors generate the forces that drive microtubule bundle expansion and bipolar motors, such as Kinesin-5 oppose this process. This work provides an important bridge between molecular dynamic simulations that focus on the behavior of individual motors and macroscopic analysis that treats the underlying geometry of axons abstractly. Athamneh and Suter first provide a brief overview of neuronal mechanics and then directly tackle the question of why reported values of neuronal forces vary over five orders of magnitude (i.e., from 0.001 to 100 nN). They suggest that a reasonable explanation for this wide degree of variation is that different types of neurons and regions of neurons naturally generate different levels of forces and that the size and mechanical properties of the probe may have an influence on what is and can be measured. In addition, they make the important point that standardized calibration methods are needed to ensure that force measurements are consistent between studies. Altogether, these studies illustrate the importance of bridging molecular, computational, and mechanical analysis for understanding neuronal mechanics.

## Summary

Over the past decades there has been an explosion in our knowledge of the molecules that regulate neuronal and glial growth and morphology. At this point, the major molecular players appear to be well-established, and the primary questions center on how they work together to create the physical manifestation of the nervous system. A major technical challenge in the field is to investigate mechanical properties of neurons and relate them to biochemical phenomena. The articles published in this research topic provide an interdisciplinary forum for a new branch of neuroscience dedicated to understanding neuronal mechanics and transport.

## Author contributions

All authors listed, have made substantial, direct and intellectual contribution to the work, and approved it for publication.

## Funding

This work was supported by the following grants: NSF 1146944-IOS (to DS), NSF 0951019-IOS, and NIH 1R01MH094607-01A1 (to KM).

### Conflict of interest statement

The authors declare that the research was conducted in the absence of any commercial or financial relationships that could be construed as a potential conflict of interest.

